# Serum sCD163 Levels Are Associated with Type 2 Diabetes Mellitus and Are Influenced by Coffee and Wine Consumption: Results of the Di@bet.es Study

**DOI:** 10.1371/journal.pone.0101250

**Published:** 2014-06-30

**Authors:** Gemma Rojo-Martínez, Elsa Maymó-Masip, M. Mar Rodríguez, Esther Solano, Albert Goday, Federico Soriguer, Sergio Valdés, Felipe Javier Chaves, Elías Delgado, Natalia Colomo, Pilar Hernández, Joan Vendrell, Matilde R. Chacón

**Affiliations:** 1 UGCI de Endocrinología y Nutrición, Instituto de Biomedicina de Málaga (IBIMA), Hospital Regional Universitario, Málaga, Spain; 2 Centro de Investigación Biomédica en Red de Diabetes y Enfermedades Metabólicas CIBERDEM, Barcelona, Spain; 3 Hospital Universitari de Tarragona Joan XXIII, IISPV, Universitat Rovira i Virgili, Tarragona, Spain; 4 CIBERObn Fisiopatología, Obesidad y Nutrición, Institut D’investigacio Biomedica De Girona Dr Josep Trueta, Girona, Spain; 5 Department of Endocrinology and Nutrition Hospital del Mar, Parc de Salut Mar, Universitat Autónoma de Barcelona, Barcelona, Spain; 6 Genotyping and Genetic Diagnosis Unit, Fundación de Investigación del Hospital Clínico de Valencia-INCLIVA, Valencia, Spain; 7 Departamento de Medicina-Endocrinología y Nutrición, Hospital Universitario Central de Asturias (HUCA), Universidad de Oviedo, Oviedo, Spain; University of North Carolina at Chapel Hill, United States of America

## Abstract

**Objective:**

Serum levels of soluble TNF-like weak inducer of apoptosis (sTWEAK) and its scavenger receptor CD163 (sCD163) have been linked to insulin resistance. We analysed the usefulness of these cytokines as biomarkers of type 2 diabetes in a Spanish cohort, together with their relationship to food consumption in the setting of the Di@bet.es study.

**Research Design and Methods:**

This is a cross-sectional, matched case-control study of 514 type 2 diabetes subjects and 517 controls with a Normal Oral Glucose Tolerance Test (NOGTT), using data from the Di@bet.es study. Study variables included clinical and demographic structured survey, food frequency questionnaire and physical examination. Serum concentrations of sTWEAK and sCD163 were measured by ELISA. Linear regression analysis determined which variables were related to sTWEAK and sCD163 levels. Logistic regression analysis was used to estimate odd ratios of presenting type 2 diabetes.

**Results:**

sCD163 concentrations and sCD163/sTWEAK ratio were 11.0% and 15.0% higher, respectively, (P<0.001) in type 2 diabetes than in controls**.** Following adjustment for various confounders, the OR for presenting type 2 diabetes in subjects in the highest *vs* the lowest tertile of sCD163 was [(OR), 2,01 (95%CI, 1,46–2,97); *P* for trend <0.001]. Coffee and red wine consumption was negatively associated with serum levels of sCD163 (*P* = 0.0001 and; *P* = 0.002 for coffee and red wine intake, respectively).

**Conclusions:**

High circulating levels of sCD163 are associated with type 2 diabetes in the Spanish population. The association between coffee and red wine intake and these biomarkers deserves further study to confirm its potential role in type 2 diabetes.

## Introduction

Common diabetes risk factors offer limited ability to identify subjects at risk of developing type 2 diabetes mellitus. Consequently, cross sectional epidemiological studies are being undertaken to identify new biomarkers to estimate better this risk. Classically, inflammatory markers have been one of the most widely explored in different cohorts, but results to date are far from conclusive [1].

Recently, the inflammatory imbalance between pro-inflammatory and anti-inflammatory molecules is gaining attention as an approach to interpret a specific metabolic scenario in which a chronic low inflammatory environment is a typical event [2] [3]. Tumor necrosis factor weak inducer of apoptosis (TWEAK), a cytokine member of the TNF- family, has emerged as a Janus-faced molecule, functioning as a pro-inflammatory cytokine in autoimmune diseases [4] [5], but with anti-inflammatory behavior in many high cardiovascular (CV) risk morbidities, such as type 2 diabetes [3] [6].

TWEAK has two protein forms, a full-length form which contains the transmembrane domain (mTWEAK), and a smaller secreted soluble form that is released into the circulation after proteolytic cleavage by a furin (sTWEAK) [7] [8]. Reduced circulating sTWEAK levels have been demonstrated in patients with type 2 diabetes suffering from chronic renal failure, and in subjects with increased atherosclerosis or type 1 diabetes [9] [10] [11] [12]. Recently, in a prospective follow-up study of a high CV risk cohort, we found that sTWEAK appears to have predictive value as a marker in the development of type 2 diabetes, highlighting a possible pathogenic role of sTWEAK in this disease [13] [14].

Circulating levels of sTWEAK could be modulated, in part, by a scavenger receptor termed CD163 (Cluster of Differentiation 163). This protein, encoded by the CD163 gene, is a member of the scavenger receptor cysteine-rich (SRCR) superfamily, and is exclusively expressed in monocytes and macrophages. CD163 is also released as a soluble form in the circulation (sCD163). It has been postulated that macrophages can recognize and internalize sTWEAK, thereby decreasing its plasma concentration [15]; although this is controversial [16]. High circulating levels of sCD163 have been linked with a more pro-inflammatory profile since it has recently been associated with long-term occurrence of type 2 diabetes in a Danish cohort, underscoring its role as a potential biomarker of diabetes [17].

Dietary-induced weight loss has been shown to modify circulating levels of sCD163 in obese patients [18]. Also, it is well known that the adherence to a healthy diet, such as Mediterranean diet and certain food consumption, are associated with an improvement in the inflammatory environment and a reduced risk of diabetes, both in prospective [19] and intervention studies [20] [21].

In view of these observations, we hypothesized that the chronic inflammatory imbalance in patients with type 2 diabetes may be linked to sCD163 and/or sTWEAK levels and in turn, this might be influenced by the type of food consumption.

We have used a wide-population based cross-sectional Spanish survey to test the concurrence of differences in circulating levels of these proteins in subjects with type 2 diabetes, exploring the influence of food consumption on plasma level variability.

## Research Design and Methods

### Study population

Cross-sectional, case-control study of 514 type 2 diabetes subjects and 517 controls (normal an oral glucose tolerance test (NOGTT), namely fasting glucose <110 mg/dL and post OGTT glucose <140 mg/dL) matched by sex, age and BMI, using data from the Di@bet.es study [22]. The Di@bet.es study was a nation-wide, cross-sectional, population-based survey conducted between 2009 and 2010. A cluster sampling design (100 health centers randomly selected with a probability proportional to population size) was used to select participants, forming a representative random sample of the Spanish population. For the analysis presented here, all diabetic subjects from the original Di@bet.es study were selected and matched with controls selected from the same population [23].

The study was approved by the Ethics and Clinical Investigation Committee of Hospital Regional Universitario (Malaga, Spain) in addition to other regional ethics and clinical investigation committees throughout Spain, and written informed consent was obtained from all participants.

### Variables and procedures

Participants were invited by mail and/or telephone to attend one examination visit at their health center. Information was collected using an interviewer-administered structured questionnaire, followed by a physical examination by a nurse. Fasting blood sampling and OGTT were performed.

Socio-demographic data collected included age, sex, smoking status (current smoker, ex-smoker or never smoker), and personal history of diabetes and hypertension.

Weight, height and waist and hip circumferences were measured by standardized methods [19]. The body mass index (BMI) and waist-to-hip ratio (WHR) were calculated as weight/height^2^ (kg/m^2^) and waist/hip (both in cm), respectively.

Blood pressure was measured using a blood pressure monitor (Hem-703C, Omron, Barcelona, Spain) following the manufacturer’s instructions. Blood pressure was measured with the subject seated and after 5 minutes of rest. Two readings were obtained and the mean value was used in the analyses.

A qualitative food frequency questionnaire (FFQ) was administered face-to-face by a trained dietitian [24]. Annual frequency consumption of 50 food items were specified in 11 different categories as follows: never/seldom, 1 and 2–3 times/month, 1, 2–3 and 4–6 times/week and 1, 2, 3, 4 and >4 times/day. For this study, food items in the FFQ were grouped in 34 categories (Table 1) and the type of oil used for either dressing, cooking, frying or making mayonnaise was queried.

**Table 1:** Clinical, anthropometrical and food habits of the study groups.

	T2DM	NOGTT		*P*
n	514	517		–
Age	64.9±11.7	64.9±11.8		0.9
% Men	54.5	54.5		1
BMI (kg/m^2^)	30.2±4.4	30.1±4.4		0.6
WHR	0.97±0.08	0.94±0.08		0.0001
SBP (mmHg)	146.4±20.0	141.2±18.4		0.0001
DBP (mmHg)	81.1±10.6	79.6±9.4		0.07
Smoking (%)	16.8	15.9		0.7
Treatment with antidiabetic drugs and/or insulin	61.6	–		–
Treatment with lipid-lowering drugs (%)	35.2	23.8		0.001
**Serum concentrations** * **:**				
sTWEAK (pg/mL)	645.7 (391.0–1254.2)	665.1 (425.0–1242.7)		0.8**
sCD163 (ng/mL )	181.5 (148.1–221.7)	163.5 (131.2–200.9)		0.0001**
sCD163/sTWEAK index	0.23 (0.12–0.45)	0.20 (0.12–0.37)		0.001**
HOMA-IR index	3.2 (1.9–5.1)	1.8 (1.3–2.6)		0.0001**
CRP (mg/L)	2.8 (1.3–5.2)	2.1 (1.1–4.1)		0.01**
Cholesterol mg/dL	192 (168–222)	204 (179–228 )		0.0001***
HDL-Cholesterol mg/dL	45 (38–54)	48 (42–57)		0.0001***
Triglycerides mg/dL	136 (98–202)	111 (84–146)		0.0001***
LDL-Cholesterol mg/dL	102 (82–122)	109 (93–131)		0.0001***
**Percentage (%) of people who take a serving at least once a day of:**				
Breakfast cereals	2.8	5.2		0.06
White bread	80.4	84.3		0.1
Whole bread	19.4	18.6		0.8
Sugar, honey, candy, jam and similar	25.2	67.7		<0.0001
Chocolate	6.4	12.7		0.001
Cakes, muffins and similar	27.5	36.4		0.002
Eggs	13.1	10.7		0.2
Dairy	63.7	68.2		0.1
Milkshake, pudding, custard, ice cream	6	6.2		0.8
Butter, cream, lard	3	3.2		0.9
Margarine	9.2	6.4		0.1
Salads (raw vegetables)	38.1	38.3		0.9
Vegetables as a main course or side dish	10.3	9.3		0.6
Stews with some vegetables	8.6	6.7		0.3
Potatoes	8.2	10.8		0.1
Fresh fruit and juices	81.1	84.7		0.1
Nuts	8.8	13.1		0.03
Snacks and appetizers	0.9	1.5		0.5
Meat	15.9	16.1		0.9
Sausage	14.6	11.2		0.1
Red wine	22.5	23.3		0.7
Other types of wine	5.2	6.6		0.3
Beer	9.9	11.8		0.3
Liqueurs and spirits	2.8	2.3		0.5
Any type of alcoholic beverage	33.9	39.5		0.05
Sugared soft drinks	8.2	8.4		0.9
Sugar free soda	8.4	3.8		0.002
Caffeinated coffee	41.8	48.4		0.03
Decaffeinated coffee	38.6	32.3		0.03
Tea	5.4	5.1		0.8
**Percentage (%) of people who take a serving at least twice a week of:**				
Rice, Italian pasta and similar	19.4	18.7		0.8
Pulses (lentils, beans, chickpeas)	6.4	6.9		0.7
Fresh fish and seafood	15.5	20		0.06
Canned fish and seafood	7.9	6.7		0.4
**Percentage (%) of people who take always olive oil for:**				
Seasoning	96.6	97.2		0.7
Cooking	91.4	91.9		0.8
Frying	73	76		0.2
Mayonnaise	50	49.9		0.9

*Data are median (IQR).

**Adjusted by WHR and SBP.

***Adjusted by WHR, SBP and lipid-lowering drugs treatment.

**T2DM;** Type 2 diabetes mellitus**; NOGTT,** Normal oral glucose tolerance test; **BMI**; Body mass Index**; WHR**; Waist to hip ratio; **SBP**; Systolic Blood Pressure, **DBP**; Diastolic Blood pressure; **HOMA-IR** homeostatic model assessment insulin resistance index; **CRP**, C-reactive protein.

Subjects with baseline capillary blood glucose levels lower than 7.8 mmol/L (140 mg/dL) and those not currently receiving treatment for diabetes underwent a standard OGTT. A venous blood sample was taken from each subject (overnight fast and post load). Samples were immediately centrifuged and stored at −18°C (15 days maximum) until shipment to the centralized CIBERDEM Biobank, where the samples were stored at −80°C for later analysis.

Serum glucose, triglycerides, and cholesterol were measured enzymatically, and HDL-Cholesterol by a direct method on an Artchitect C8000 Analyzer (Abott Laboratories SA, Madrid, Spain). Serum insulin was measured by immunochemiluminescence on an Architect I8000 Analyzer (Abbott Laboratories SA). The homeostatic model assessment insulin resistance index (HOMA-IR) was calculated by the formula: (serum glucose (mmol/L) x serum insulin (mU/L))/22.5.

The diagnosis and classification of diabetes was based on plasma glucose results, using the 1999 WHO criteria [25].

Obesity was considered to be a BMI ≥30 kg/m^2^, and abdominal obesity a waist circumference >102 cm in men or >88 cm in women.

Hypertension was defined as ongoing antihypertensive treatment or systolic blood pressure ≥140 mmHg and/or diastolic blood pressure ≥90 mmHg.

Serum concentrations of sTWEAK and sCD163 were determined in duplicate by ELISA using the commercially available, human TWEAK/TNFSF12 kit #DY1090, and human CD163 kit #DY1607 (R&D Systems Europe, Abingdon, Oxon, UK), respectively. The intra- and inter-assay CVs were 2.5% and 7.0% for sTWEAK, and 2.4% and 6.4% for sCD163, respectively. The sensitivity of the test was of 62 pg/mL for sTWEAK and 1.56 ng/mL for sCD163.

Plasma concentrations of high-sensitivity C-reactive protein (CRP) was determined by a highly sensitive immunonephelometry kit (Dade Behring, Marburg, Germany). CRP cut-off value to exclude acute inflammation was 10 mg/L.

### Statistical analysis

Statistical analysis was performed using the SPSS/PC statistical package (version 19 for Windows; SPSS, Chicago, IL). For clinical and anthropometric variables, normal distributed data was expressed as mean value and SD, and for variables with no Gaussian distribution, values were expressed as median (25–75^th^ percentile). For statistical analysis, values of variables that did not have a Gaussian distribution were logarithmically transformed.

The hypothesis testing for continuous variables was performed using t-test (or the Mann-Whitney test in the event of non-normality of distributions). Associations between the qualitative characteristics were tested by χ^2^ test. Linear regression analysis was used to determine what variables were related to sTWEAK and sCD163 serum concentrations.

Logistic regression analysis was used to estimate odd ratios of presenting type 2 diabetes. Results were expressed as standardized coefficient (β) and 95% confidence interval for β [95% CI(B)]. To evaluate dose-response relationships, *P* values for trend were calculated. In all cases the level of rejection of a null hypothesis was α<0.05.

## Results

The characteristics of the studied population are shown in Table 1. Groups were similar in age, sex ratio and BMI, however type 2 diabetes patients had higher WHR and blood pressure. Smoking habit was similar in both groups. Type 2 diabetes mellitus patients were treated with anti-diabetic drugs and/or insulin in 61.6% of cases and lipid-lowering drugs were taken by 35.2% of diabetes patients and 23.8% of NOGTT subjects. No differences were found in serum sTWEAK levels between study groups, but serum sCD163 levels, sCD163/sTWEAK index, C-reactive protein (CRP) and HOMA-IR were increased in patients with type 2 diabetes in 11.0%, 15.0%, 33.3% and 77.7% respectively, after adjusting for WHR and systolic blood pressure. Serum lipids were different between studied groups even when adjusted by WHR, SBP and lipid-lowering drug treatment.

The association between the presence of diabetes and the frequency of intake of selected food groups was also analysed (Table 1). People with type 2 diabetes often take less cereals, sugars (honey, candy, jam and similar), chocolate, cakes, nuts, alcoholic beverages, sugared soft drinks and caffeinated coffee in general and, take more often decaffeinated coffee and sugar free soft drinks.

Spearman bivariate correlation analysis with the entire studied population showed that sTWEAK, sCD163 and sCD163/sTWEAK index were weakly and significantly related with fasting glucose (r = −0.08, *P*<0.01; r = 0.13, *P*<0.001; and r = 0.11, *P*<0.01 respectively); WHR (r = −0.07, *P*<0.01; r = 0.06, *P*<0.01; r = 0.1, *P*<0.001, respectively), and HOMA-IR (r = −0.06, *P*<0.01; r = 0.21, *P*<0.001; r = 0.14, *P*<0.001; respectively), although relations were direct with sCD163 and sCD163/sTWEAK index, and reverse with sTWEAK. Additionally, a weak but significantly positive relation was found between sCD163 and BMI (r = 0.09, P<0.001).

Levels of sTWEAK, sCD163 and sCD163/sTWEAK index were also analyzed in relation to food intake. The intake of each food item was coded into two categories, as shown in Table 1. Univariate analysis showed that sTWEAK levels were different according to the intake of cereals, margarine and caffeinated coffee (*P*<0.05, data not shown). Levels of sCD163 were different depending on the intake of olive oil, sweets, cakes, meat, canned fish, wine, coffee, eggs, butter, vegetables and legumes (*P*<0.05, data not shown). The sCD163/sTWEAK index was found associated to foods in a similar manner to sCD163.

Regression analysis showed that, after adjusting for several confounding variables, caffeinated coffee and red wine intake were the only foods that remained associated solely with sCD163 (β = −0.062, *P* = 0.05 and β = −0.108, *P* = 0.001 respectively, Table 2). The association between the sCD163/sTWEAK index and HOMA-IR remained in the adjusted models (β = 0.096, *P* = 0.009, Table 2), in contrast to that of sTWEAK or sCD163 (Table 2).

**Table 2:** Relationship of sCD163, sTWEAK and sCD163/sTWEAK index with caffeinated coffee and red wine.

	Log sTWEAK		Log sCD163		Log sCD163/sTWEAK index	
	Standarizedβ coefficient	p	Standarizedβ coefficient	p	Standarizedβ coefficient	p
**Sex**	0.054	0.1	0.077	0.04	–0.015	0.6
**Age**	–0.018	0.6	0.020	0.5	0.019	0.5
**Coffee intake**	–0.024	0.4	–0.062	0.05	–0.031	0.3
**Red wine intake**	–0.013	0.6	–0.108	0.001	–0.036	0.2
**WHR**	–0.036	0.3	0.072	0.05	0.061	0.1
**SBP**	0.001	0.9	–0.008	0.8	–0.007	0.8
**Diabetes presence**	–0.001	0.9	0.094	0.006	0.067	0.05
**Log_HOMA-IR**	–0.050	0.1	0.054	0.1	0.096	0.009
**Log_CRP**	0.002	0.9	0.06	0.06	0.02	0.5
**R^2^ of the adjusted model**	0.01	0.3	0.06	0.001	0.03	0.001

Interaction between type 2 diabetes and variables shown in Table 2 was not significant, thus the major associations with the included variables were reported in Table 3. We found that sTWEAK levels were not different according to sex, BMI, abdominal obesity or high blood pressure presence; neither were they different with respect to coffee, decaffeinated coffee or red wine intake, but sTWEAK levels were moderately increased in non-smoking subjects.

**Table 3:** Serum concentrations of sTWEAK. sCD163 and sCD163/sTWEAK index according to characteristics of the studied subjects.

	TWEAK pg/ml	CD163 ng/ml	sCD163/sTWEAK index
**Men**	603.8 (392.8–1236.7)	166.7 (133.2–205.7)	0.21(0.13–0.41)
**Women**	726.5(429.5–1266.6)	179.2(144.6–216.1)	0.22(0.13–0.41)
***p***	0.1	0.001	0.8
**BMI<30**	663.0(404.3–1247.4)	167.8(134.3–203.9)	0.21(0.13–0.40)
**BMI≥30**	648.2(407.5–1251.4)	179.6(142.3–220.0)	0.22(0.13–0.43)
***P***	0.9	0.0001	0.07
**Abdominal obesity (sex adjusted)**			
**No**	585.9(400.6–1238.3)	163.5(129.8–199.6)	0.21(0.13–0.39)
**Yes**	693.5(411.4–1251.4)	180.4(145.5–220.6)	0.22(0.13–0.43)
***p***	0.3	0.0001	0.04
**High blood pressure**	612.4(406.0–1232.9)	166.3(133.2–203.0)	0.22(0.13–0.40)
**No high blood pressure**	672.1(405.1–1254.7)	175.5(139.5–213.5)	0.22(0.13–0.42)
***p***	0.6	0.01	0.5
**Smoking**	610.2(392.2–1160.8)	162.0(126.7–196.6)	0.21(0.13–0.40)
**No smoking**	673.3(409.7–1258.6)	175.2(140.4–213.1)	0.22(0.13–0.41)
***p***	0.05	0.001	0.7
**Coffee less than one cup per day**	788.2(406.6–1290.1)	179.4(142.3–221.1)	0.21(0.13–0.41)
**Coffee at least one cup per day**	578.0(404.9–1196.5)	166.0(133.0–203.1)	0.22(0.13–0.41)
***p***	0.07	0.0001	0.9
**Decaffeinated coffee less than one cup per day**	665.2(406.3–1266.3)	170.4(138.0–208.4)	0.21(0.13–0.41)
**Decaffeinated coffee one or more cups per day**	661.2(404.3–1237.7)	177.2(139.1–216.3)	0.24(0.13–0.41)
***p***	0.1	0.1	0.7
**Red wine less than one drink per day**	658.7(410.9–1255.2)	175.4(140.6–215.9)	0.23(0.13–0.41)
**Red wine one or more drinks per day**	703.2(383.7–1242.5)	164.0(129.7–201.2)	0.19(0.13–0.40)
***p***	0.5	0.002	0.07

However, sCD163 was significantly increased in women, in subjects with BMI ≥30, abdominal obesity, high blood pressure and with active smoking status, and decreased in subjects taking caffeinated coffee or red wine at least once per day. The sCD163/sTWEAK index was increased in people with abdominal obesity, but did not change with any other variable.

In the multivariate analysis, to calculate the likelihood of having diabetes adjusted by abdominal obesity, high blood pressure, and coffee and red wine intake (logistic regression, Table 4), subjects with serum values of sCD163 in tertile 3 had twice the risk of diabetes compared with subjects with serum values in the lower tertile 1 (*P* for trend ≤0.0001). However, lower circulating levels of sTWEAK were not associated with the presence of diabetes (*P* for trend = 0.1), but sTWEAK tertile 2 with respect to tertile 1 were found associated; this data is not considered of importance since tertile 3 versus tertile 1 is not associated. Smoking did not alter the results (data not shown).

**Table 4:** Likelihood of having diabetes (T2DM = 1; control = 0) according to selected variables of interest.

			95% CI of OR		
	*P*- value	OR	Low	High	*P*-for trend
**One or more cups of caffeinated coffee per day**	0.1	1.19	0.91	1.56	
**One or more drinks of red wine per day**	0.3	1.17	0.86	1.59	
**sTWEAK tertile 1 (<448** **pg/mL. reference)**		1			
**sTWEAK tertile 2 (449–1040** **pg/mL)**	0.04	0.72	0.52	0.99	0.1
**sTWEAK tertile 3 (>1041** **pg/mL)**	0.1	0.77	0.56	1.06	
**sCD163 tertile 1 (<149** **ng/mL. reference)**		1			
**sCD163 tertile 2 (150–197** **ng/mL)**	0.008	1.52	1.11	2.09	≤0.001
**sCD163 tertile 3 (>197** **ng/mL)**	0.0001	2.01	1.46	2.77	
**Abdominal obesity**	0.0001	2.06	1.58	2.69	
**High blood pressure**	0.0001	1.71	1.26	2.31	

CI: confidence interval; OR: odds ratio.

## Discussion

In this study we report that circulating levels of sCD163 are associated with type 2 diabetes in a Spanish population and these levels may be influenced by coffee and red wine consumption.

Several epidemiological studies have associated inflammation to type 2 diabetes [26] [27]. Elevated levels of clinical markers and inflammation mediators may be reduced after intensive life-style modifications. Furthermore, experimental data showing a link between insulin resistance and different inflammatory candidates have suggested that inflammation is intimately involved in the pathogenesis of type 2 diabetes. Accordingly, the search for useful serum biomarkers remains a major focus in many epidemiological studies. Recently, circulating soluble levels of sCD163 have been linked to long-term onset of type 2 diabetes in a Danish cohort, proposing its clinical utility as a biomarker of diabetes [17]. Previous data on obesity, a condition usually linked with insulin-resistance and low chronic inflammation, revealed that circulating levels of sCD163 mirrored particular inflammatory markers such as CRP and TNFα [28]. Consistent with these findings, a close relationship between sCD163 and BMI, WHR and HOMA-IR was demonstrated in our study cohort. Furthermore, a positive association with CRP was detected also in our study. Thus, high circulating levels of sCD163 were associated with a higher prevalence of type 2 diabetes in this Spanish population. Nevertheless, sTWEAK levels were not linked with a protective cardiovascular profile in this study. Generally, studies conducted in high CV risk populations indicate that low levels of sTWEAK may point to a worse scenario [11]. A recent study highlighted the relevance of decreased serum sTWEAK as a predictive marker of type 2 diabetes. Interestingly this study was conducted in a high CV risk population, in which the incidence of type 2 diabetes was assessed during a follow up [13]. In this large prospective nested case-control study, lower sTWEAK serum levels were found in incident cases compared to matched controls. Indeed, previous cross-sectional studies have also proposed a link between sTWEAK concentration and type 2 diabetes [10]. However, in our case-control study, we did not find a clear association of sTWEAK and the presence of type 2 diabetes. Differences between studies could be explained by differences in cohort definitions. Our subjects came from a random general population without bias for any special cardiovascular risk factor. This may have reduced the power of sTWEAK as a useful biomarker, as was proposed in previous reports. Of note, we cannot discard a potential effect of this cytokine in type 2 diabetes because of the inherent limitation of a cross-sectional design. We are aware that a prospective follow-up study would be more appropriate to unequivocally confer or deny a role in type 2 diabetes occurrence, but the data presented in this work tempers the usefulness of sTWEAK as a biomarker of type 2 diabetes in a general population.

With respect to dietary habits, we have observed a strong inverse relationship between sCD163 levels and some foods typically associated with a reduced risk of diabetes, such as coffee and red wine. Although we did not find a direct association between sCD163 and type 2 diabetes, it is well known that diet might have important effects on mediators of inflammation [29] [30]. Consequently, our results provide the first indication for an inverse relationship between coffee and red wine and circulating levels of sCD163. These data are compatible with the available reports describing the beneficial effect of moderate consumption of coffee and wine in delaying the onset of type 2 diabetes; however, no causal effect can be inferred from the study design. [31]. Epidemiologic studies have consistently linked coffee consumption with a lower risk of type 2 diabetes [32]. In human trials, consumption of eight cups of coffee per day significantly decreases plasma levels of proinflammatory cytokines such as IL-18, together with significant increases in adiponectin levels [33]. Similarly, coffee consumption has been inversely associated with markers of inflammation and endothelial dysfunction both in healthy women and those with type 2 diabetes [34]. Experimental data in a mouse model of diabetes also confirm a suppressive effect of coffee consumption on hyperglycemia, by improving insulin sensitivity in part due to reducing inflammatory cytokine expression and decreasing fat liver accumulation [35]. Caffeine has multiple actions on physiology, including inhibition of the DNA damage response, and its metabolites 1-methylxanthine and 1-methyluric acid are potent antioxidants [36]. However, the effects of caffeine on sCD163 release have not yet been investigated.

Equally, a negative association was also observed between sCD163 levels and red wine consumption. A recent study has shown that the Mediterranean diet, rich in polyphenols, reduces the incidence of type 2 diabetes [37]. In vitro, wine polyphenols, particularly the flavonoids procianidins from grape reduces expression of pro-inflammatory cytokines such as MCP-1, IL-6 and TNF-α by inhibiting NF-κB nuclear translocation [38]. Notwithstanding the results observed, we should highlight that the frequency nutritional survey was not quantitative; therefore, we cannot measure whether these effects are due to the amount of caffeine or other active compounds in foods.

We found reduced levels of circulating sTWEAK and sCD163 in smokers. These data corroborate our previous findings on sTWEAK in smokers [13]. The potential influence of smoking on sTWEAK levels appears to be a complex issue that remains to be fully elucidated. Unexpectedly, levels of sCD163 were also found to be reduced in smokers compared with non-smokers. Data concerning the effect of smoking on inflammation appears to be contradictory; whilst cigarette smoke has been associated with oxidative stress and an inflammatory response [39], others have reported that nicotine, a major constituent of tobacco smoke, exerts anti-inflammatory effects on different cell types and appears to be beneficial in disorders where inflammation-related mechanisms are involved, including ulcerative colitis and obesity [40] [41].

In summary, the data presented here provide new evidence for the participation of sCD163/sTWEAK binomial on the prevalence of type 2 diabetes in the general population. Furthermore, food consumption may influence the circulatory cytokine profile related to sCD163, as coffee and red wine associated with a more anti-inflammatory profile. New prospective studies are required to confirm the pathogenic link between these associations.

## Acknowledgments

We wish to acknowledge the kind collaboration of the people who voluntarily participated in the study, and to the Di@bet.es study Steering Committee that is formed by the following researchers: Federico Soriguer (coordinator), Luis Castaño, Albert Goday, Miguel Catalá, Alfonso Calle-Pascual, Gemma Rojo-Martínez, Ramón Gomís, Rafael Carmena, Manuel Serrano-Rios, Alfonso López-Alba, Inmaculada Mora-Peces, Conxa Castell, Elias Delgado, Edelmiro Menéndez, Josep Franch, José A. Vázquez, Sonia Gaztambide, Juan Girbés, Sergio Valdés, Emilio Ortega, Roser Casamitjana and Joan Vendrell.
